# Galectin-like protein 2: A new non-mammalian member of the galectin family

**DOI:** 10.1016/j.psj.2026.107952

**Published:** 2026-10-11

**Authors:** Juliane Günther, Antonio Di Maio, Alexander Rebl, Ralf Middendorff, Yan Liu, Sebastian P. Galuska

**Affiliations:** a Research Institute for Farm Animal Biology (FBN), Wilhelm-Stahl-Allee 2, Dummerstorf, 18196, Germany; b Glycosciences Laboratory, Faculty of Medicine, Imperial College London, Du Cane Rd, London, W12 0NN, United Kingdom; c Institute of Anatomy & Cell Biology, Faculty of Medicine, Justus-Liebig-University, Aulweg 123, Giessen, 35392, Germany

**Keywords:** Galectin-like, Glycan, Testis, Epididymis, Seminiferous fluid

## Abstract

The galectin family comprises evolutionarily conserved soluble animal lectins. Recently, a new member of this family was described: galectin-like 2 (LGALSL2). This protein consists of a globular domain that shares the structural organization of galectin carbohydrate recognition domains, but lacks some of the amino acids necessary for binding to β-galactosides. Although LGALSL2 is widespread in jawed vertebrates, this protein is absent in mammals. The amino acid sequence is highly conserved from cartilaginous fish to birds. The focus of this conservation is on the convex face of the protein, which is located opposite the putative glycan-binding groove. In chickens, LGALSL2 is predominantly expressed in the testis and epididymis. We demonstrated that recombinant chicken LGALSL2 protein binds to vesicle structures in the Sertoli cell cytoplasm as well as in epididymal ductuli efferentes proximales vesicles. The location of these vesicles indicates their potential role in the formation and resorption of the seminiferous fluid. These vesicle structures are both rich in carbohydrates. However, broad-spectrum glycan microarray screening revealed that, under the tested conditions, LGALSL2 did not show significant binding to the glycan sequences, with only very weak signals observed for to a few fucosylated glycans. The results suggest that LGALSL2 does not have a classic galectin function. Instead, it may recognise 'exotic' glycans not represented in the screening array, or its activity may depend less on glycan-protein interactions and more on protein-protein interactions. As these binding partners of LGALSL2 appear to be involved in the formation and turnover of seminiferous tubular fluid, it is suggested that this new galectin-like protein may be involved in the maturation of sperm in chickens.

## Introduction

Galectins belong to a family of carbohydrate-binding proteins found in animals. These small proteins evolved into a large group with a wide range of functions in bilaterians (Cummings, et al., 2022; Zhang, et al., 2025). The evolution of vertebrates has led to an explosive expansion of the galectin family. Classic galectins in Gnathostomata have the ability to bind to β-galactosides via a conserved carbohydrate recognition domain (CRD). The sugar-binding groove in the CRD of galectins comprises five subsites (A–E) (Chan, et al., 2018). Subsites C and D are highly conserved within galectins. Subsite C binds β-galactose, while subsite D binds either glucose (in the case of the disaccharide lactose) or *N*-acetylglucosamine (in the case of *N*-acetylglucosamine). Subsites A, B and E are more variable, binding either sugars or functional groups that are adjacent to β-galactose (subsites A and B) or glucose or *N*-acetylglucosamine (subsite E). It is these three subsites that define the sugar specificity of individual galectins. Thus, sugars that bind to subsites A and B of some galectins can increase binding affinity to respective glycans, whereas in others, they can decrease or completely prevent it. Galectin 1, for instance, tolerates α1,2-linked fucose and α2,3-linked sialic acid glycans. In contrast, the closely related Galectin 2 only tolerates glycans with α1,2-linked fucose, but not sialylated glycans (Stowell, et al., 2008). Galectins are categorized as either prototypical or chimeric with one CRD, or tandem-repeat with two CRDs connected by a linker region. Their common origin probably lies in a tandem-repeat galectin found in the proto-vertebrate ancestor. This has multiplied through two genome duplications that occurred during vertebrate evolution, as well as gene duplications combined with translocations (Günther and Galuska, 2023). In addition to classic galectins, there are galectin-like proteins (LGALSL) and Grifin. These proteins can be classified as galectins based on gene and protein structure, as well as through synteny studies. However, key amino acids have been altered in these proteins, causing them to lose their ability to bind β-galactosides. Alongside the long-known LGALSL (also known as HSPC159) and Grifin proteins, we recently classified the LGALSL2 protein as belonging to the galectin family (Günther and Galuska, 2023). LGALSL2 is present in all Gnathostomata groups, ranging from cartilaginous fish to terrestrial vertebrates; however, it was lost during mammalian evolution. Nevertheless, the gene is found in the other branch of amniotes, which includes modern reptiles and birds.

Birds are a class of endothermic amniotes that comprises over 11,000 extant species. The chicken (*Gallus gallus*) is a widely studied bird species that is of particular importance in agriculture and has been a key model organism in developmental biology and phylogenetic studies for several decades.

As no studies on LGALSL2 have yet been conducted, in this study we took a closer look at this galectin-related protein in chickens. Our focus here is on one of the main sites of LGALSL2 expression in chickens: the testis and epididymis.

## Materials and methods

### Protein structure analysis and molecular modelling

LGALSL2 proteins from all gnathostome groups were identified via syntenic genes (Vti1b, Zfyve26 and Plek2, (Günther and Galuska, 2023) in the NCBI Gene and Genome databases (https://www.ncbi.nlm.nih.gov/gene/ and https://www.ncbi.nlm.nih.gov/genome/gdv/). An amino acid sequence comparison was performed by aligning the sequences and conducting a phylogenetic analysis using MEGA X (Kumar, et al., 2018). The structure of the chicken LGALSL2 protein was predicted using the Robetta algorithm (Baek, et al., 2021). The protein structure and conserved amino acids of LGALSL2 were visualized using Chimera X 1.8 (Meng, et al., 2023). The consensus sequence was determined using an entropy-based measurement in AL2CO (Pei and Grishin, 2001) and independent count weighting within the Chimera X program.

### LGALSL2 cloning and expression

The bacterial vector pGEX-6P-1, which is used to express GST fusion proteins, was modified to allow the recombinant expression of the LGALSL2 protein. An additional 6xHis-tag and an Avi-tag, which enables targeted biotinylation of the protein, were introduced into the vector using in vitro mutagenesis (Q5 Site-Directed Mutagenesis Kit, New England Biolabs GmbH, Frankfurt am Main, Germany). This was performed sequentially according to the manufacturer's instructions, using the following primers (respective insertions are indicated by lowercase letters): caccatcacCTGGGATCCCCGGAATTC (His-tag forward), atggtgatgGGGCCCCTGGAACAGAAC (His-tag reverse), atttttgaagcgcagaaaattgaatggcatgaaCTGGGATCCCCGGAATTC (Avi-tag forward), atcgttcaggccgctttgaaaataaagattttcGTGATGGTGATGGTGATGG (Avi-tag reverse). The Avi-tag primers were used to introduce an additional TEV cleavage site, allowing the GST and His-tag to be enzymatically removed. The resulting expression vector enables the recombinant expression of proteins in *E. coli* with an N-terminal tag of the following structure: GST – prescission cleavage site – 6xHis – TEV cleavage site – Avi-tag.

To clone the LGALSL2 protein, RNA was first isolated from rooster testis tissue using the innuPREP RNA Mini Kit 2.0 (IST Innuscreen GmbH, Berlin, Germany), before being transcribed into cDNA using the SCRIPT cDNA Synthesis Kit (Jena Bioscience GmbH, Jena, Germany). The LGALSL2 sequence was amplified using Q5 High-Fidelity DNA Polymerase (New England Biolabs) and the following primers, each of which contains a necessary restriction site for cloning: ACGCGTCGACTGACAGAGAAGGAGAGGTGTC (forward, SalI site underlined) and ATAGTTTAGCGGCCGCTCAAGCCACTTTGGTAAGCTTG (reverse, NotI site underlined). The PCR product was cleaved with *Sal*I and *Not*I (Life Technologies GmbH, Darmstadt, Germany), ligated into the expression vector (Rapid DNA Ligation Kit, Life Technologies GmbH), and then transformed into *E. coli* (NEB 10-beta Competent *E. coli*, New England Biolabs). The vector was verified by sequencing (Microsynth AG, Göttingen, Germany).

The LGALSL2 vector was transformed into the AVB101 *E. coli* B strain (Avidity, LLC, Aurora, Colorado, USA) for recombinant expression. This strain contains a biotin ligase expression vector (pBirAcm). Expression with simultaneous in vivo biotinylation was performed overnight at room temperature according to the manufacturer's instructions. The *E. coli* culture was then harvested, centrifugated and the pellet was stored at -20°C.

### Purification of the recombinant biotinylated LGALSL2 protein

The bacterial pellet was lysed using an ultrasonic homogenizer (LABSONIC® M, Sartorius, Göttingen, Germany) on ice with lysis buffer (125 mM Tris-HCl, pH 7.5; 150 mM NaCl; 1 mM EDTA; 1 mM DTT; 1 mg/ml lysozyme), to which PMSF and BenzNuclease (Benz-Neburase, GenScript Biotech, Rijswijk, Netherlands) were added. The lysate was then centrifuged at 13,000 rpm for 30 minutes at 4°C. The recombinant LGALSL2 protein in the supernatant was then bound to PureCube Glutathione MagBeads (Cube Biotech, Monheim, Germany). Following subsequent washing steps using lysis buffer without lysozyme, the biotinylated LGALSL2 protein was eluted from the beads using His-tagged TEV protease (GenScript Biotech). The TEV protease was removed by incubating the eluate with PureCube Ni-NTA MagBeads (Cube Biotech). The purified LGALSL2 protein was stored at 4°C in 40 mM Tris-HCl (pH 7.5), 500 mM NaCl, 0.4 mM EDTA, 0.8 mM DTT and 10% glycerol.

### Galectin qRT-PCR analysis

Eleven-week-old roosters were slaughtered at our local abattoir (EU approval ES1635), in compliance with all relevant ethical and legal requirements. As the animals were killed as part of normal slaughtering procedures for food production without any animal experiments being carried out, no special ethical approval was required. The testis and epididymis were removed, minced, shock-frozen in liquid nitrogen and stored at -70°C. RNA extraction and cDNA synthesis were performed as described above. PCR primers were designed for all chicken galectins and the reference genes *RPL30, RPL37A, PGK2, PPIA, EIF2B1* and *TBP* (Table 1). All primer pairs exhibited efficiencies between 92.7% and 109.5%. Standard plasmids were obtained by cloning the PCR products amplified with the designed primer pairs into TOPO PCR cloning vectors (Life Technologies GmbH) and verifying the inserts by sequencing. These plasmids were then used to generate standard curves (R^2^ > 0.99; 10^7^–10^2^ copies per 5 µL) for subsequent normalization of the qRT PCR data.

**Table 1:** Oligonucleotide primers used for qRT-PCR.

Gene	Name	Oligonucleotide primers (sense and antisense)	Amplicon length [bp]	Primer efficiency [%]
*RPL30*	ribosomal protein L30	TTCGGCAAGGCAAAGCCAAGTT	173	n.d.
		AGCCAGCGTGCACACTCTGTAG		
*RPL37A*	ribosomal protein L37a	TGGTACCCGTTATGGCGCATCC	165	n.d.
		CAGGCACCACCAGCAACTGTCT		
*PGK2*	phosphoglycerate kinase 2	AATCCTGCAAACGGCTCCGTCA	191	n.d.
		TGAGCACGATGGGCAGTTCCAA		
*PPIA*	peptidylprolyl isomerase A	GTTCTTCGACATCGCCGCCAAC	156	n.d.
		GGGATGATCCGGTGGAAGCAGG		
*EIF2B1*	translation initiation factor eIF-2B subunit alpha	AGCCAGACCAAGCAGGGCAAAA	199	n.d.
		TGAGCCTTGGCACACACAGCAA		
*TBP*	TATA-box binding protein	CTTTGCCGGGAACCACACCTCT	172	n.d.
		CGGGCACGAAGTGCAATGGTTT		
*LGALS1A*	galectin 1A	GCGCCTCACTGTCAAGGGGATA	171	101.82
		TGCTCGGTACCCCACTCTTCCA		
*LGALS1B*	galectin 1B	CTCGCTTTGACTGCCATGGGGA	199	92.67
		ATGCCCAGCCGATTGGGAAACT		
*LGALS2*	galectin 2	TCTGACAACTGGCAGCAGGAGC	154	101.91
		TGTCATAGCCATGCCGGTTGGG		
*LGALS3*	galectin 3	CGTACTCTGAAGCTCCAGCCGC	156	102.21
		AGGCAATGTCTTGCCCCCTCTT		
*LGALS8*	galectin 8	GGTGGCCTTGTTCCTGGAGAGC	151	109.52
		AGCCAGACCATTTGAAGCGGGG		
*LGALSL*	galectin-like	GGGACGCGCCGAAAATAGAGGA	195	95.87
		CACAGGTCAGGCTGATGCCGAA		
*LGALSL2*	galectin-like 2	GCGGACTGAGACCTGCCATGAG	183	95.95
		TGCCCCACTTCCCATCAATTCGT		

The qRT-PCR analysis of three testis and epididymis samples were performed using the LightCycler 96 system (Roche) with a final reaction volume of 12 µl, consisting of 6 µl SensiFAST SYBR No-ROX Mix (Bioline/Meridian Bioscience), 5 µl cDNA and 1 µl primer mix. The LightCycler protocol included an initial denaturation step at 95°C for 5 minutes, followed by 40 cycles of denaturation at 95°C for 5 s, annealing at 60°C for 15 s and elongation at 72°C for 15 s. Fluorescence was recorded at 72°C for 10 s during each cycle. Melting curve analysis was conducted to confirm amplification specificity and the absence of non-specific products. Amplicon sizes, ranging from 151-199 bp (Table 1), were verified by electrophoresis on 2% agarose gels containing ethidium bromide and visualized using a ChemiDoc MP imaging system (Bio-Rad). qPCR data were extracted as Cq values using the LightCycler-96 analysis software v. 1.1.0.1320 (Roche). Individual copy numbers were calculated based on gene-specific standard curves and normalized using a normalization factor derived from the Cq values of RPL37A, which was selected from six candidate reference genes based on coefficient of variation (CV) values below 25%. Differences in gene expression between testes and epididymis were analyzed using a paired t-test, accounting for paired measurements from the same individuals.

### Carbohydrate histochemistry on paraffin-embedded tissue sections

Tissue from the testes and epididymis of a one-year-old rooster was fixed in Modified Davidson's Solution for one day, followed by embedding in paraffin. Five-micrometer-thick sections were prepared using a microtome. After rehydration of the slides, the collagen fibers were visualized using a Masson-Goldner trichrome staining kit (Morphisto, Offenbach am Main, Germany). Carbohydrates were visualized by periodic acid-Schiff staining using a PAS staining kit (Carl Roth, Germany). The microscopic images were captured using a Zeiss Axio Imager A1 microscope (Carl Zeiss Microscopy Deutschland GmbH, Oberkochen, Germany).

### LGALSL2 histochemistry

Recombinant biotin-labelled LGALSL2 was prepared as described above. Rehydrated slides were boiled in citrate buffer pH 6.0, and then blocked in PBS buffer containing 1% BSA and 0.1% Tween 20. This was followed by an overnight incubation at 4°C with LGALSL2 in the same buffer. Binding was detected using streptavidin Alexa Fluor 555 (AF555). Control slides were incubated with streptavidin AF555 only. Cell nuclei were stained with SYBR Green.

### Lectin histochemistry

For lectin histochemistry, the slides were rehydrated and boiled in citrate buffer pH 6.0. The slides were then blocked in HEPES buffer (10 mM HEPES, pH 7.5, 150 mM NaCl, 0.1 mM CaCl_2_, 0.01 mM MnCl_2_) with the addition of 1% BSA and 0.1% Tween 20 for one hour at room temperature. The slides were then incubated with the biotinylated lectins peanut agglutinin (PNA) and *Aleuria aurantia* lectin (AAL) (Vector Laboratories, Inc., Newark, California, USA) in the same buffer overnight at 4°C. To verify sugar-specific binding, control slides were incubated in parallel with either PNA and 200 mM galactose or AAL and 400 mM L-fucose. To detect specific binding to sialic acids, control slides were incubated with α2-3,6,8 neuraminidase (New England Biolabs) overnight at 37°C prior to the blocking step. The control slides and slides not pretreated with neuraminidase were then incubated overnight at 4°C in HEPES buffer containing biotinylated *Sambucus nigra* lectin (SNA) or *Maackia amurensis* lectin II (MAL II) (both from Vector Laboratories). Binding of all four lectins was detected using streptavidin AF555 (MoBiTec, Hamburg, Germany) conjugate. The cell nuclei were stained with SYBR Green. All fluorescence microscopic images were captured using a Zeiss LSM800 Axio Observer Z1 microscope (Carl Zeiss Microscopy Deutschland GmbH). The images were analyzed using Zeiss Zen 3.9 software.

### Glycan microarray screening

The binding specificity of the biotinylated LGALSL2 protein was assessed using a broad spectrum neoglycolipid (NGL)-based, sequence-defined glycan screening array comprising 672 lipid-linked oligosaccharide probes of both mammalian and non-mammalian origin, essentially as described previously (Lundstrøm, et al., 2024; Shahin, et al., 2023). The full list of probes and their sequences is provided in Supplementary Table S1.

Microarray analyses were carried out largely as reported earlier (Liu, et al., 2012). Briefly, microarray slides were blocked for 1 h with a BSA-based blocker/diluent solution prepared in 10 mM HEPES (pH 7.4), 150 mM NaCl, 1% (w/v) bovine serum albumin (Sigma 7030), 0.02% casein blocker (Pierce), and 5 mM CaCl₂. The biotinylated LGALSL2 protein was incubated on the slides for 90 minutes at a concentration of 20 µg/mL, followed by a detection step which involve a 30-minute overlay of streptavidin–Alexa Fluor 647 (Molecular Probes) at 1 µg/mL. All steps were conducted at room temperature. Additional details regarding the glycan library, microarray fabrication, imaging procedures, and data analysis are provided in the Supplementary Glycan Microarray document (Supplementary Table S2), in accordance with the Minimum Information Required for A Glycomics Experiment (MIRAGE) guidelines for reporting glycan microarray data (Liu, et al., 2017). The quality control experiment with biotinylated AAL (Vector B1395, tested at 2 µg/mL) was performed under the same conditions as described above.

## Results

### Structural properties of galectin-like protein 2

Structural modelling shows that LGALSL2 has the typical galectin structure, consisting of β-sheets that create two surfaces. The S1-S2 sheets and the loops in between form a groove on the S-face (Fig. 1). This groove provides the binding region for carbohydrates in classical galectins. In contrast, the antiparallel surface, consisting of the F1–F5 sheets, forms a convex surface. In galectins, this F-face usually interacts with protein binding partners. The large loop between the S3 and S4 sheets of LGALSL2 is notable. This loop is significantly smaller in other galectin domains that correspond to CRD2. An exception is the galectin-like protein LGALSL, also known as HSPC159. Moreover, of all the galectins, this LGALSL has the closest amino acid sequence similarity to the LGALSL2 protein. The S3-S4 loop is also highly variable in LGALSL2 proteins within the Gnathostomata groups. Examining the amino acid conservation within the LGALSL2 proteins reveals that the F-face exhibits the highest level of conservation (Fig. 1). In contrast, the S-face hardly shows any conservation. Comparing the calculated secondary structure of chicken LGALSL2 with that of human galectin 9′s CRD2 reveals an identical β-sheet structure (Fig. 2). However, the absence of some key amino acids in the LGALSL2 S4, S5 and S6 β-sheets, which mediate galectin binding to lactose or N-acetylglucosamine, is striking. This is particularly evident in the S4 β-sheet. In contrast, the key amino acids in the S5 and S6 sheets appear to be mostly present. For instance, the W residue that mediates the CH-π interaction with the galactose ring in galectins is present in all analyzed LGALSL2 proteins.

**Fig. 1:**
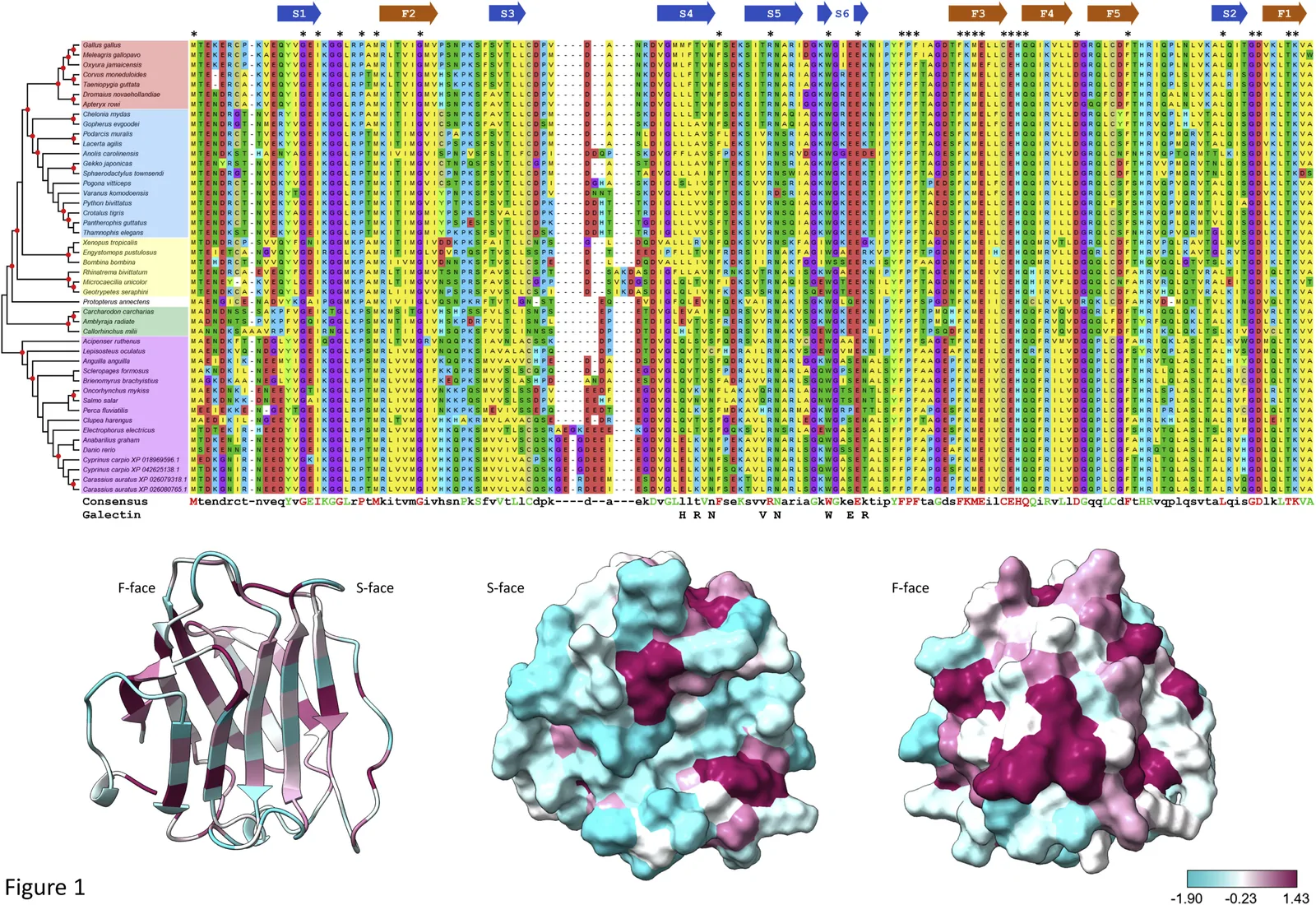
Structure and sequence homology of the chicken galectin-like 2 protein. (A) Sequence alignment of LGALSL2 proteins from Aves (red box), Reptilia (blue box), Amphibia (yellow box), Osteichthyes (purple box) and Chondrichthyes (green box). Amino acids are labeled according to the MegaX color code. The positions of the β-strands (S1–S6 and F1–F6) are indicated by blue and orange arrow, respectively, above the alignment. Evolutionary history was inferred using maximum likelihood and a JTT matrix-based model (Jones, et al., 1992). The tree with the highest log-likelihood (-4668.42) is shown on the left. Branches marked with red circles indicate that more than 75% of the associated taxa are clustered together. The initial tree for the heuristic search were obtained automatically by applying the Neighbour-Join and BioNJ algorithms to a matrix of pairwise distances estimated using the JTT model, and then selecting the topology with the superior log likelihood value. This analysis included 46 amino acid sequences. There were a total of 154 positions in the final data set. The consensus sequence shown below the alignment was obtained using an entropy-based measure in AL2CO and independent counts weighting. Conserved amino acids are indicated by colored capital letters (100% conservation red; >80% conservation green). The positions of the corresponding amino acids critical for binding lactose/N-acetyl-lactosamine in classical galectins are shown in the bottom row. (B) Protein structure of the chicken LGALSL2 protein, as predicted using the Robetta algorithm. The ribbon diagram and the surfaces of the S- and F-faces of the protein are colored to show sequence conservation, from least conserved in cyan, to white, to most conserved in marron.

**Fig. 2:**
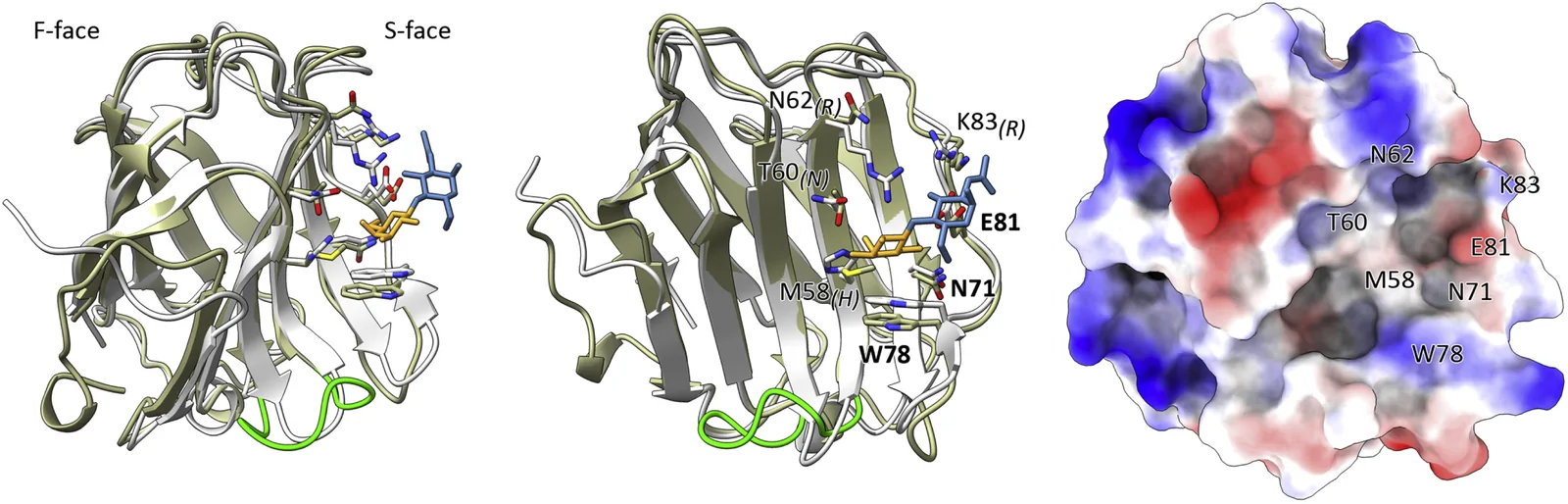
Comparison of predicted structure of chicken LGALSL2 with the C-terminal CRD of human galectin 9 in complex with N-acetyllactosamine (pdb: 3nv2). The ribbon diagram of LGALSL2 is colored gold and those of the human galectin 9 is silver. The large S3-S4 loop, which is typical of LGALSL2 and LGALSL proteins, is highlighted in green. The amino acids involved in galectin-specific N-acetyllactosamine/lactose binding and the corresponding amino acids in LGALSL2 are shown. Amino acids in LGALSL2 are given using one-letter code. If they are different from those in galectin 9, these are listed in brackets. Amino acids that are identical are displayed in bold font. The calculated coulombic surface of chicken LGALSL2 indicates the electrostatic potential of the potential glycan ligand binding groove on the S-face.

### Galectin expression in chicken testis and epididymis

To identify the tissues in which *LGALSL2* is expressed in chickens, we searched the RNA-Seq exon coverage data for chicken tissues available in the NCBI database. According to these data, *LGALSL2* is most highly expressed in testis tissue (NCBI BioSample database (http://www.ncbi.nlm.nih.gov/biosample/) accession number SAMEA103992290). As the testis and epididymis are both necessary for maturation of sperm, we analyzed the expression of all chicken galectins in these tissues using qRT-PCR (Fig. 3). We found that all genes coding for galectins and the two galectin-like proteins in chickens are expressed in both the testes and the epididymis; however, *LGALSL* is expressed at a very low level. Interestingly, most galectin genes are expressed more strongly in the epididymis than in the testis. *LGALS2* and *LGALSL2* are exceptions, as these two genes are significantly more highly expressed in the testis. *LGALSL* is expressed at a comparably low level in both tissues.

**Fig. 3:**
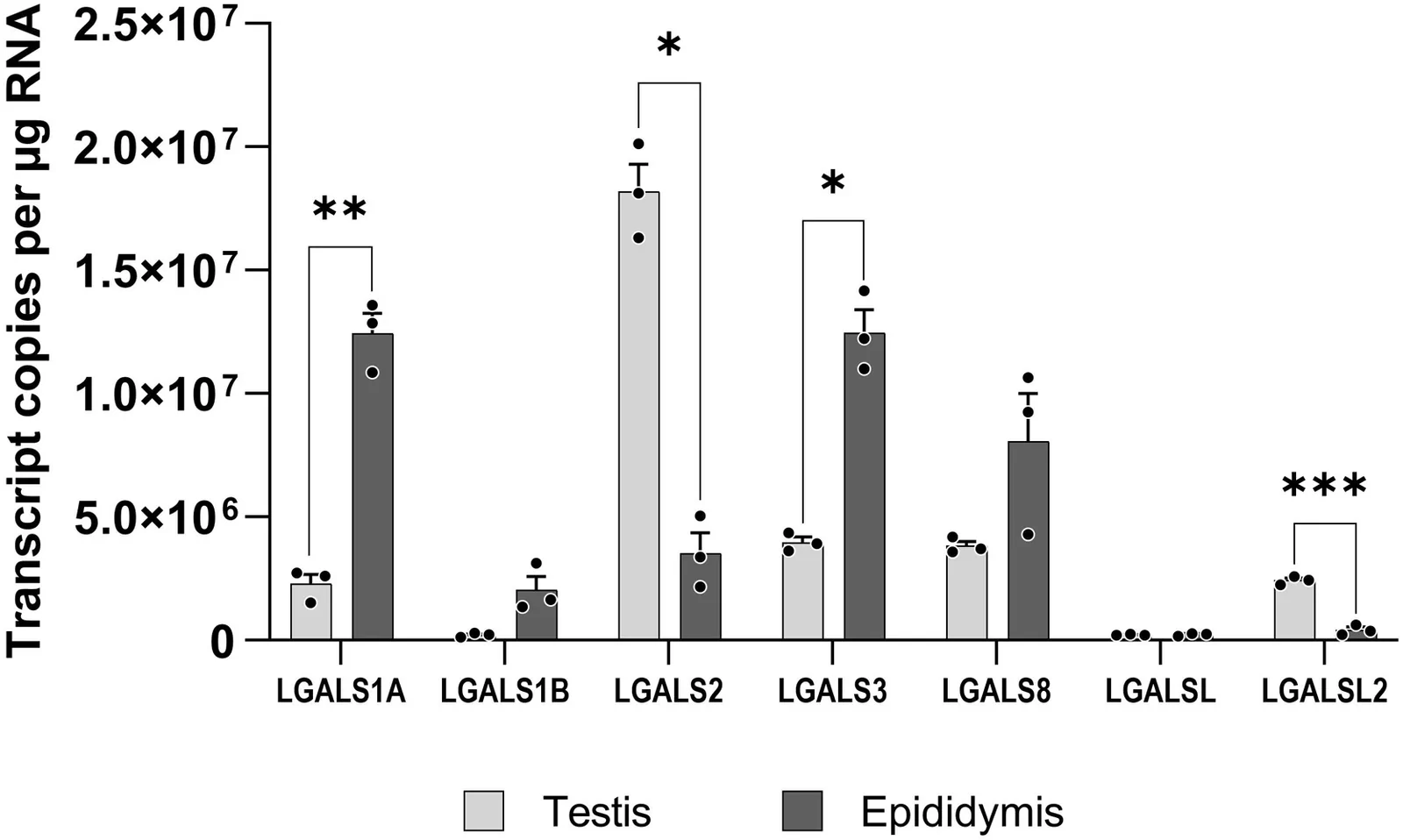
Galectin transcript levels in testis and epididymis of eleven weeks old chicken (n = 3). The levels of all transcripts encoding galectin and galectin-like proteins found in the chicken genome are expressed copies per µg total RNA. The chart displays the mean value + SEM of the transcripts in the testis (black bar) and epididymis (grey bar). Asterisks represent significantly different transcript levels between testis and epididymis (*, p < 0.05; **, p < 0.01; ****, p < 0.001).

### LGALSL2 binding sites in testis and epididymis

In order to determine whether there are specific binding regions for LGALSL2 in the testis and epididymis, the LGALSL2 protein was recombinantly expressed in *E. coli*. The presence of an N-terminal Avi-tag allowed for in vivo biotinylation during expression. The protein was then purified using a GST-tag, which was removed when the purified protein was eluted. Tissue slides were incubated with biotin-labelled LGALSL2, after which the binding was detected using fluorophore-labelled streptavidin (Fig. 4). Weak binding was observed on the fibers of the lamina propria surrounding the seminiferous tubules. A significantly stronger binding signal was detected in the Sertoli cell vesicles of the testis (Fig. 4D) and the ductuli efferentes proximales (Dep) epithelium (Fig. 4E) of the epididymis. No binding was evident on the rete testis (Rt) (not shown) or the epithelium of the distal ducts [ductuli conjugentes (Dc) and ductus epididymidis (De)] (Figs. 4B and E).

**Fig. 4:**
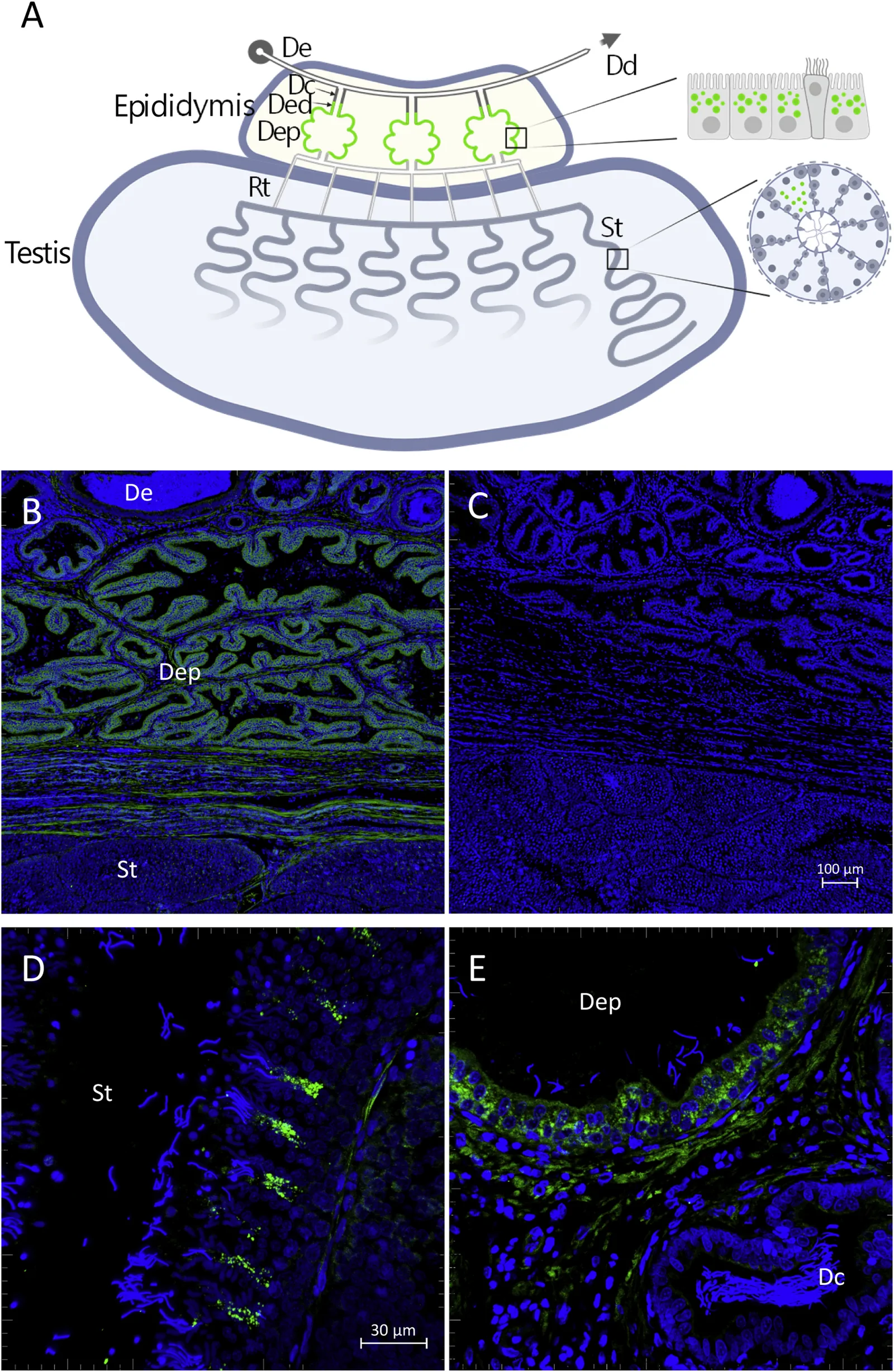
LGALSL2 galectin histochemistry of the testis and epididymis. (A) Schematic illustration of testis and epididymis of the rooster. The image illustrates the sperm-producing seminiferous tubules (St) of the testis and the compartments responsible for sperm transport and maturation within the epididymis. The rete testis (Rt), ductuli efferentes proximales (Dep; the convoluted sack-like structure is indicated schematically) and distales (Ded), ductuli conjugentes (Dc), ductus epididymidis (De), and ductus deferens (Dd) are shown in sequence. The diagrams on the right show cross-sections of a seminiferous tubule and the epithelium of the Dep. The vesicles in the Sertoli cells and those in the non-ciliated epithelial cells of the Dep are highlighted in green. See Supplementary Figure S1 for a genuine representation of the testis and epididymis using PAS and Masson's trichrome staining. (B-D) Binding of LGALSL2 to the testis and epididymis. Paraffin-embedded tissue slides were incubated with biotin-tagged LGALSL2 protein. LGALSL2 binding was detected using AF555-tagged streptavidin (green). The nuclei were counterstained with SYBR Green (blue). (A) Overview of LGALSL2 binding to the testes and epididymis. There is spot staining of cells in the St and extensive staining of the Dep epithelium and testis capsule. Testis and epididymis interstitial tissue shows weaker staining. De is not stained. The diagram was created with BioRender.com. (B) Control incubation of the tissue slides with streptavidin-AF555 reveals nuclear counterstaining only. (C) A close-up view of a seminiferous tubule shows LGALSL2 binding to vesicles in the Sertoli cell cytoplasm. (D) A close-up view of the epididymis reveals LGALSL2 binding to vesicles in the cytoplasm of Dep epithelial cells and connective tissue fibers in the epididymal interstitium.

To analyze whether the vesicles in Sertoli cells and the proximal epididymal epithelium, to which LGALSL2 binds, contain carbohydrates, the tissue was stained with PAS (Fig. 5, Supplementary Figure S1B). We observed numerous PAS-positive vesicles in the cytoplasm of Sertoli cells, and strong PAS staining appeared on the apical border of Dep epithelial cells and in the vesicles in their cytoplasm. In contrast, the epithelial cells of the Rt (Supplementary figure S1B) and Dc/De (Fig. 5C) do not exhibit any carbohydrate-rich structures. Additionally, the collagen fibers of the testicular capsule, lamina propria, and interstitial tissue (Figure S1A) showed strong PAS staining (Figure S1B).

**Fig. 5:**
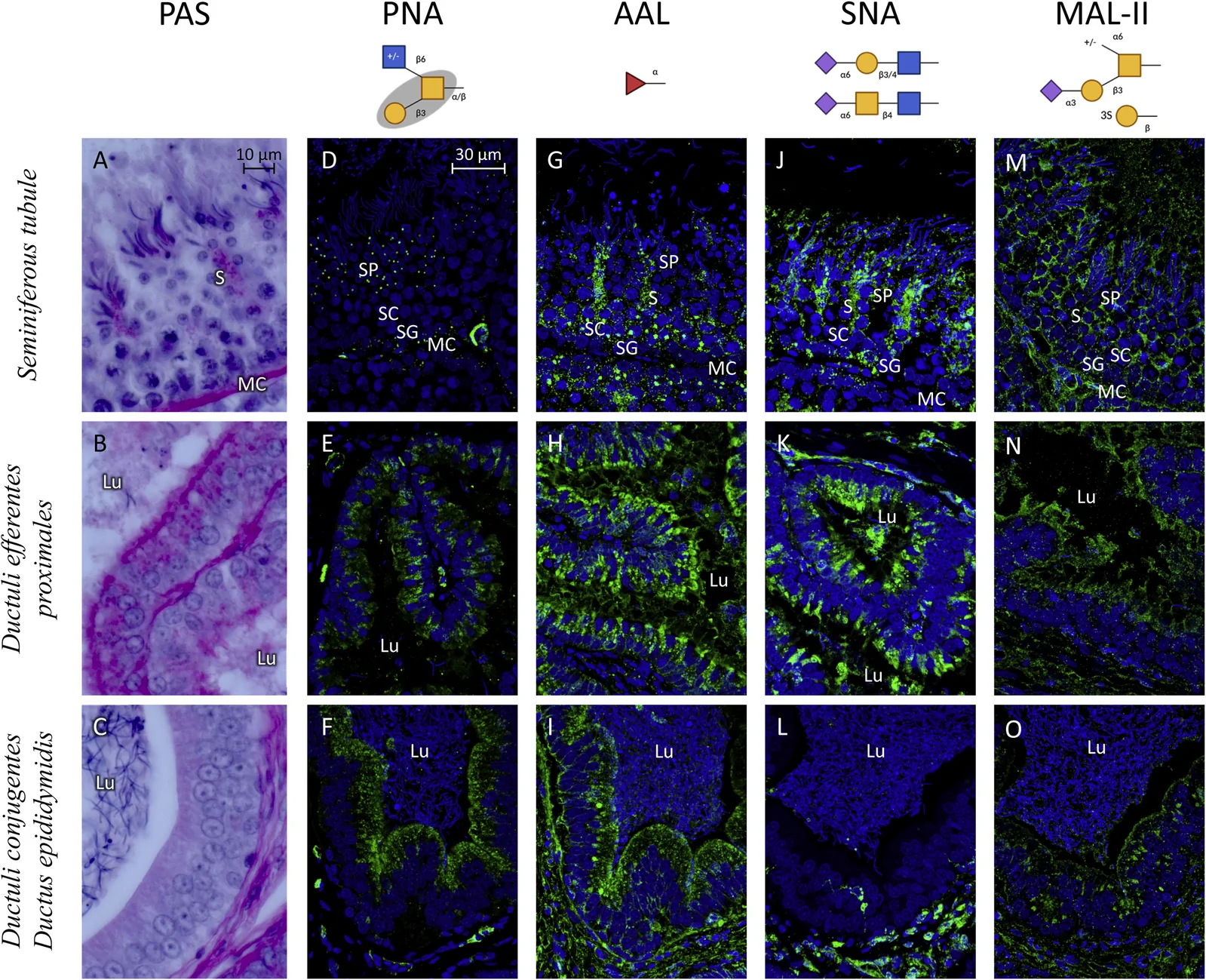
PAS staining and lectin histochemistry of seminiferous tubules, D. efferentes proximales, and D. conjugetes/D. epididymidis. (A-C) PAS staining; (D-F) PNA, (G-I) AAL, (J-L) SNA, and (M-O) MAL-II binding. The main glycan-binding motifs of the lectins PNA, AAL, SNA and MAL-II (Bojar, et al., 2022) are illustrated using the Symbol Nomenclature for Glycans (SNFG). See Supplementary Figure S2 for an overview of the different lectin stainings of the testis and epididymis, as well as the control slides. Lu, lumen; MC, myoid cell; S, Sertoli cell; SC, spermatocyte; SG, spermatogonium; SP, spermatid.

Since the carbohydrate-binding groove of galectins is known to possess subsites that bind additional sugars linked to the core β-galactoside, thereby determining the specificity of each galectin, we examined lectin binding. This technique allowed us to characterise the carbohydrates in LGALSL2-binding (PAS-positive) vesicles in detail. The lectins used detect the T antigen (Galβ1,3GalNac) [peanut agglutinin (PNA)], as well as glycans with terminal α-linked fucose [*Aleuria aurantia* lectin (AAL)] and α2,6- and α2,3-linked sialic acids [*Sambucus nigra* agglutinin (SNA) and *Maackia amurensis* Iectin II (MAL-II), respectively]. In addition to its preference for α2,3-linked sialic acids containing glycans, MAL-II is also known to bind 3′O-sulfated Gal epitopes (Bojar, et al., 2022).

The distinctive PAS and LGALSL2-positive vesicle structures of Sertoli cells can be visualized using AAL and SNA (Figs. 5G and J). Additionally, AAL binds to the well-developed Golgi apparatus of spermatocytes, while SNA binds to granules/vesicles of various sizes in spermatogonia. Sertoli cell vesicles are not bound by PNA or MAL-II. PNA, however, binds to the acrosomes of spermatids and other germ cells (green vesicular structure attached to the nucleus, Fig. 5D). MAL-II signals are among the cells (Fig. 5M). Furthermore, MAL-II binds to myoepithelial cells surrounding the seminiferous tubules. The binding of PNA and AAL to testicular tissue can be completely inhibited by their respective haptenic sugars, galactose and fucose (Supplementary Figure S2B and D). Treatment with neuraminidase completely prevents subsequent SNA binding, while MAL-II binding is only inhibited in cells of the seminiferous tubule (Supplementary Figure S2F and H). Binding to interstitial cells remains unchanged.

In the Dep, PNA, AAL and SNA like LGALSL2 stain vesicles (Figs. 5E, H and K). AAL and SNA, in particular, demonstrate strong binding. MAL-II stains extracellular structures on the apical surface of the Dep epithelium (Fig. 5N). Furthermore, small vesicles can be detected in the distal epididymal ducts (Dc and De) with PNA and AAL (Figs. 5F and I). However, staining with PNA is significantly stronger and staining with AAL is significantly weaker than in the proximal epididymal duct epithelium. SNA does not bind to the D. epididymidis epithelium (Fig. 5L), and MAL-II binds only very weakly (Fig. 5O). In addition to the epithelium of the epididymal ducts, PNA binds to the endothelium of blood vessels. AAL, SNA and MAL-II also stain structure-forming connective tissue cells of the epididymal interstitium.

The binding of the lectins PNA and AAL in the epididymis can be inhibited by their respective haptenic sugars (Supplementary Figure S2B and D). Treatment with neuraminidase completely abolish the binding of SNA and only partially inhibits that of MAL-II (Supplementary Figure S2F and H). On the contrary, sialidase treatment intensifies the MAL-II staining of interstitial tissue and the luminal content of efferent ducts.

In summary, the LGALSL2-binding vesicles in Sertoli cells and in the epithelial cells of the Dep are carbohydrate-rich and can be stained with AAL and SNA. PNA, however, stains the vesicles in the Dep epithelium but not the Sertoli cell vesicles. It is important to note that, in the case of lectin binding, it is not usually just one specific glycoprotein or glycolipid that is recognized, since the same glycan can be generally present on several different glycoconjugates.

### Microarray analysis of the glycan-binding properties of LGALSL2

Since LGALSL2 binds to carbohydrate-rich vesicles in the testis and epididymis, we investigated whether this galectin-like protein exhibits glycan-binding properties despite lacking some core amino acids that are crucial for β-galactoside binding. We further aimed to define its potential glycan ligands. To this end, purified biotin-labelled LGALSL2 was used in a glycan microarray analysis. This approach enabled analysis of LGALSL2 binding across 672 glycans. The screening arrays used here have been widely used to define glycan ligands for a broad range of glycan-binding proteins derived from viral, bacterial, and plant origins, as well as mammalian immune lectins (Lundstrøm, et al., 2024; McAllister, et al., 2020; Shahin, et al., 2023; Vendele, et al., 2020). However, no significant binding was detected under the selected conditions (Fig. 6A). Only very weak signals, were observed with LGALSL2 for a small number of glycans, including fucosylated glycan probes Globo-H-Hexa and Le^b^-hexaosylceramide, and these were close to background levels (Supplementary Table S1). This contrasts with the robust signals observed with the biotinylated plant lectin AAL, which bound to almost all fucosylated glycan probes on the arrays (Fig. 6B).

**Fig. 6:**
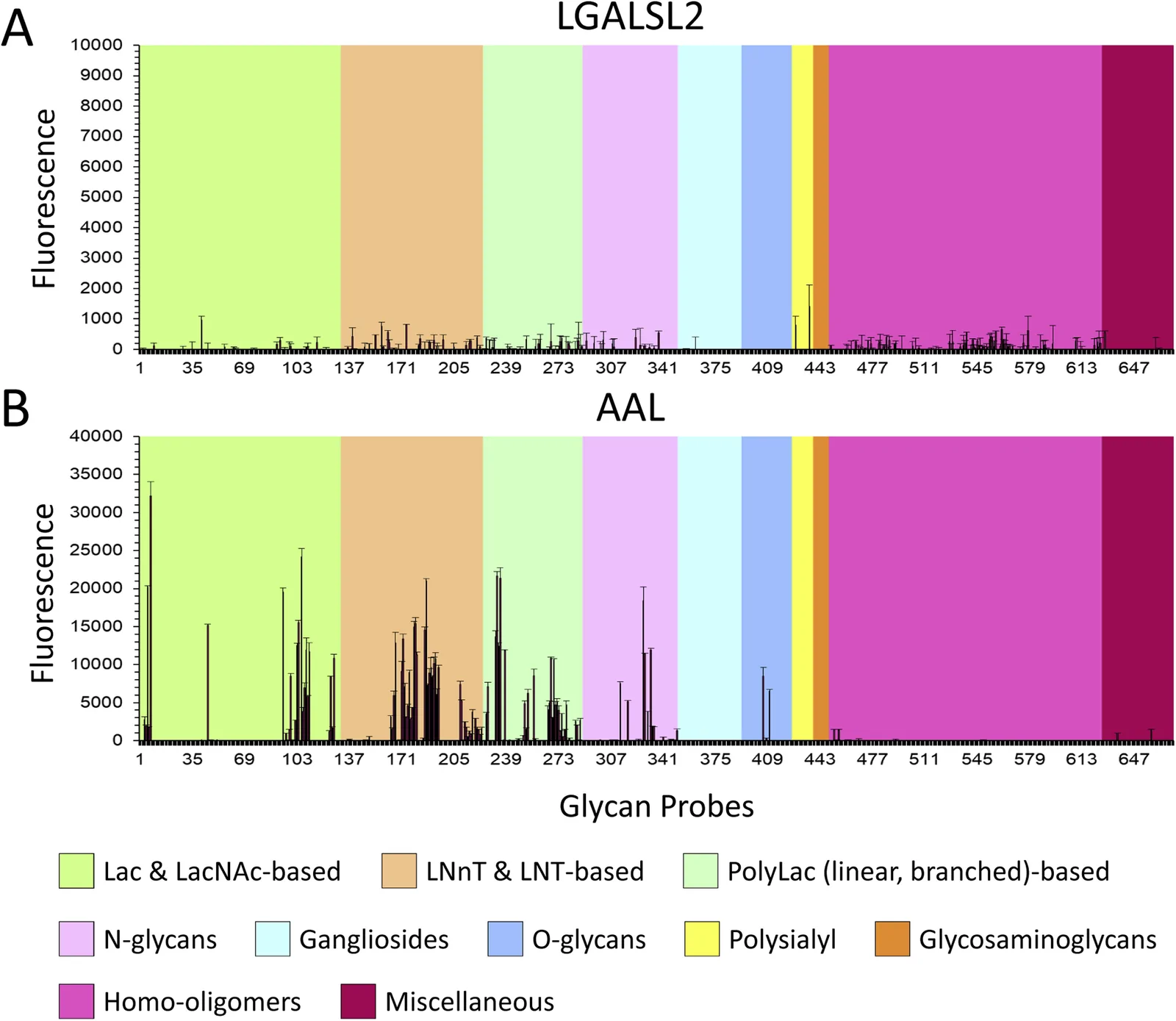
Glycan microarray binding profile of LGALSL2 compared with that of the AAL lectin. The histograms show the average fluorescence binding intensity of LGALSL2 (A) and AAL (B) to various glycan probes from two replicates, with the standard deviation indicated as error bars. The complete datasets from the array screening can be found in Supplementary Table S1.

## Discussion

Due to its synteny and the exon-intron structure of the gene, as well as the secondary structure of the protein, LGALSL2 can be clearly classified as a member of the galectin family. More specifically, it is classified as CRD2 of galectins (Günther and Galuska, 2023). Its calculated secondary structure resembles that of galectins, consisting of two antiparallel β-sheets. The S-face of LGALSL2 forms a concave groove that, in classical galectins, represents the glycan binding site. However, some of the amino acids necessary for galectin-typical β-galactoside binding are missing. Other conserved amino acids (AA) are present, however. For example, the W residue in β-sheet S5 is highly conserved within galectins. This tryptophan plays a key role in glycan-protein binding in classical galectins. It forms a CH–π interaction with the aliphatic C–H groups of the carbohydrate that is stacked on the π-electron system of the indole ring (Diehl, et al., 2024; Keys, et al., 2025). Opposite the S-face is the outwardly curved F-face in LGALSL2, which normally acts as the binding region for galectin protein binding partners (Mayo, 2023; von Hundelshausen, et al., 2021). In prototypical galectins, the region relevant for homo- or heterodimerization is found here. When LGALSL2 is compared between species, it is striking that the S-face shows very little amino acid conservation. In contrast, the F-face is significantly more conserved. This suggests that LGALSL2 proteins may exhibit high species-specific variance in possible S-face ligands, which may be glycans. Conversely, the protein interaction partners that might be involved in galectin-typical cross-linking or signal transduction and that bind to the F-face may be relatively well conserved within the Gnathostomata.

At the amino acid sequence level, LGALSL2 exhibits the greatest similarity to LGALSL (Günther and Galuska, 2023). There is also little AA conservation in the S-face of LGALSL among gnathostome species, while the F-face is highly conserved. This distinguishes both galectin-like proteins from classical galectins, for which the focus of conservation is on the S-face. Unlike LGALSL2, LGALSL is also expressed in mammals. Interestingly, starting with monotremes, the highly conserved W in the β-sheet S5 of galectins has been replaced by an arginine (García Caballero, et al., 2016). Consequently, CH–π interaction is no longer possible. This suggests that the evolution of mammals may have led to a functional change in the structurally similar LGALSL, in addition to the loss of the LGALSL2 gene.

Available NGS expression datasets from chicken tissues suggest that LGALSL2 is only significantly expressed in a few tissue types. One of these is the testis. Q-RT-PCR analyses of testis and epididymis tissue from chickens show that LGALSL2 is relatively strongly expressed in both. The other galectins examined also demonstrate significant expression levels. Notably, almost all galectin-encoding genes are expressed far more strongly in the epididymis than in the testis, except for *LGALSL2* and *LGALS2*, which were five times higher expressed in the testes than in the epididymis. The high expression of *LGALS2* in the testis is unexpected. The expression of this galectin in the male reproductive tract has not been reported in mammals, and searching transcriptomic data in public databases provides no evidence of it. Mammalian galectin 2 is primarily expressed in the gastrointestinal tract, where it plays a role in maintaining the integrity of the epithelium and the strength of the mucus layer (Negedu, et al., 2023). However, *LGALS2* appears to be expressed at a fairly high level in the testes of some non-mammalian vertebrates. This is evident in RNA-seq data from green sea turtle *Chelonia mydas* (NCBI Accession ID SAMN19133331, adult, Gonads, 17 years, male) and from *Xenopus tropicalis* testes (Accession IDs SAMN06679842 and SAMN06679843), suggesting an unknown role for galectin 2 in the reproduction of non-mammalian vertebrates.

Although qPCR can be used to determine the expression level of *LGALSL2* in the testis and epididymis, it cannot identify the specific cell types within these tissues in which it is expressed. However, a single-cell transcriptome study of the chicken testis suggests that LGALSL2 is among the up-regulated differential genes in round and elongated spermatids, as well as in mature spermatozoa (Guo, et al., 2025). For these cell types, Log₂ fold changes of 1.4, 1.7 and 0.8 are reported, respectively (see Supplementary Material 10 in Guo, et al., 2025; LGALSL2 Ensembl ID: ENSGALG00010019947). These results do not imply that *LGALSL2* is not also expressed in other testicular cells, merely that its expression level is significantly higher in spermatids and spermatozoa. Interestingly, none of the other chicken galectins are found to be up-regulated in these cells. No corresponding single-cell studies on the epididymis exist.

Histochemical binding of LGALSL2 to testicular tissue demonstrates an interaction between the galectin-like 2 protein and vesicles within Sertoli cells. These vesicles fill the Sertoli cell's cytoplasm from the basal to the apical region. They are also visible in PAS staining, indicating a carbohydrate-rich composition. In addition to the highly conserved β-galactoside-binding region, the carbohydrate-binding groove of galectins contains subsites that interact with sugars linked to the β-galactoside core structure. We therefore used lectin staining to investigate the sugars present in LGALSL2-binding vesicles. Sugars that typically bind to these subsites of mammalian galectins include galactose, fucose and sialic acid (Horlacher, et al., 2010). We therefore examined the binding of the lectins PNA, AAL, SNA, and MAL-II to the rooster's testis and epididymis in more detail. Specific lectin staining reveals that Sertoli cell vesicles can be detected using AAL and SNA, but not PNA and MAL-II. Therefore, they appear to contain glycans with a terminal α-linked fucose or α2,6-sialic acid. Sertoli cells are somatic cells in the seminiferous tubule that support spermatogenesis and nourish developing germ cells. During sperm formation, excess cytoplasmic remnants of elongated spermatids are shed as residual bodies. An important function of the Sertoli cell is to incorporate and recycle these residual bodies. The resulting phagosomes may represent the vesicles that we detected with LGALSL2. Sertoli cells also contain vesicles responsible for secreting proteins relevant to sperm fertility and forming the seminiferous tubular fluid. This route of secretion has been described in rats for prosaposin, for example (Yamamiya, et al., 2023). To our knowledge, the distinct carbohydrate content of chicken Sertoli cell vesicles, as detected by PAS and lectin staining, has not been described in mammals. PAS staining is primarily used in mammals to visualize acrosomes. However, weak SNA staining of Sertoli cell cytoplasm has been observed in cats (Desantis, et al., 2006). It is possible that the composition of Sertoli vesicles differs between chickens and mammals. A notable feature of chickens is that their sperm achieve a high degree of maturity in the seminiferous tubules. They possess most of their motility when they leave the testes and are partially capable of fertilization (Howarth, 1983; Nixon, et al., 2020). This ability is then enhanced in the epididymis. This distinguishes them from mammalian sperm, which are only capable of fertilization after passing through the epididymis. One possible cause of this difference could be the secretions of the avian Sertoli cell and the seminiferous tubular fluid it produces.

The vesicles of the epithelial cells of the Dep represent another binding region for LGALSL2. No binding of LGALSL2 can be detected in the epithelia of the distal regions of the epididymis, the D. conjugentes and the D. epididymidis. The Dep vesicles are also stained with PAS and are binding sites for PNA, AAL and SNA. The strongest binding signal is observed with AAL and SNA. The tubular fluid in the Dep lumen is also strongly stained by AAL and SNA. In mammals, the main function of the ductuli efferentes is to reabsorb the seminiferous tubular fluid, including most of the luminal proteins. This process is essential for the maturation and concentration of sperm before they enter the epididymis (Hess, 2015). The predominant cells are non-ciliated cells characterized by an extensive endosomal/lysosomal apparatus. For instance, the epithelium of the efferent ducts of the Golden Syrian hamster exhibits a significant quantity of these non-ciliated cells with PAS-positive lysosomes, which increase in number from the proximal to the distal region and are absent in the caput epididymis (Ford Jr, et al., 2014). These cells are responsible for resorption and have a microvillus border that increases the surface area facing the lumen of the efferent duct. Their cell nucleus is located on the basal side. In addition, ciliated cells mix the tubular fluid and promote reabsorption. The nucleus of ciliated cells is located apically. This feature makes it easy to distinguish between the two cell types histologically. The LGALSL2 and the vesicles stained with the lectins PNA, AAL and SNA are located in the cells with a basal nucleus, i.e in the non-ciliated cells responsible for resorption.

The strong binding of LGALSL2 to Sertoli cell vesicles and endosomes/lysosomes in non-ciliated Dep epithelium cells suggests that this protein binds to compounds produced by Sertoli cells in the seminiferous tubular fluid and reabsorbed by the Dep epithelium. However, the current study does not determine the specific binding partners of LGALSL2. This requires further investigation. The tubular fluids contain a variety of glycoconjugates. The application of AAL and SNA indicates that many of the glycoconjugates contain terminal α-linked fucose or α2,6-sialic acid residues (Fig. 7). The composition of the epididymis in birds is not well understood at the glycan sequences level. Lectin histochemical studies have been conducted on the Muscovy duck (*Cairina moschata*) (Abd-Elmaksoud, et al., 2009). Ducks belong to the order Anseriformes (waterfowl), which is the sister order to Galliformes (landfowl), meaning they are quite closely related to chickens. Abd-Elmaksoud et al. detected a wide range of glycans in the efferent ducts of the Muscovy duck. They found that the efferent ducts exhibit the highest lectin binding compared to other regions of the epididymis. Unlike our results with roosters, none of the lectins tested by the authors, including those with specificity for α1-2-linked fucose (UEA-I) or Lewis x motifs (LTA) showed detectable binding to the epididymis. PNA binds strongly to the epithelium of the D. efferentes in the duck but not the D. conjugentes or D. epididymidis. Sialic acid-binding lectins were not investigated in this study. Lectin binding studies in various mammals demonstrate significant variation in glycan composition in the epididymis between species. In humans, the efferent ducts are strongly bound by AAL (AAA) and weakly by SNA, with no detectable interaction with to PNA or MAA (MAL-II), whereas the epididymis exhibit strong binding for PNA, AAL, SNA and MAL-II (Arenas, et al., 1996). In mice, PNA but not SNA has been found to bind to the efferent ducts, whereas both lectins bind to the epididymal ducts (Lohr, et al., 2010). (Gofur and Ogawa, 2024) found strong PNA binding in the epithelium of the efferent ducts of adult mice, but no UEA-I binding. Both lectins bind to areas of the epididymal ducts, with PNA staining intensity increasing toward the distal region and UEA1 staining intensity decreasing. In cats, SNA binds to the apical cytoplasm, whereas MAL-II binds to the microvilli and the Golgi apparatus of non-ciliated cells in the efferent ducts (Arrighi, et al., 2010). Thus, the binding patterns of these two sialic acid-binding lectins in cats are very similar to what we observed in roosters.

**Fig. 7:**
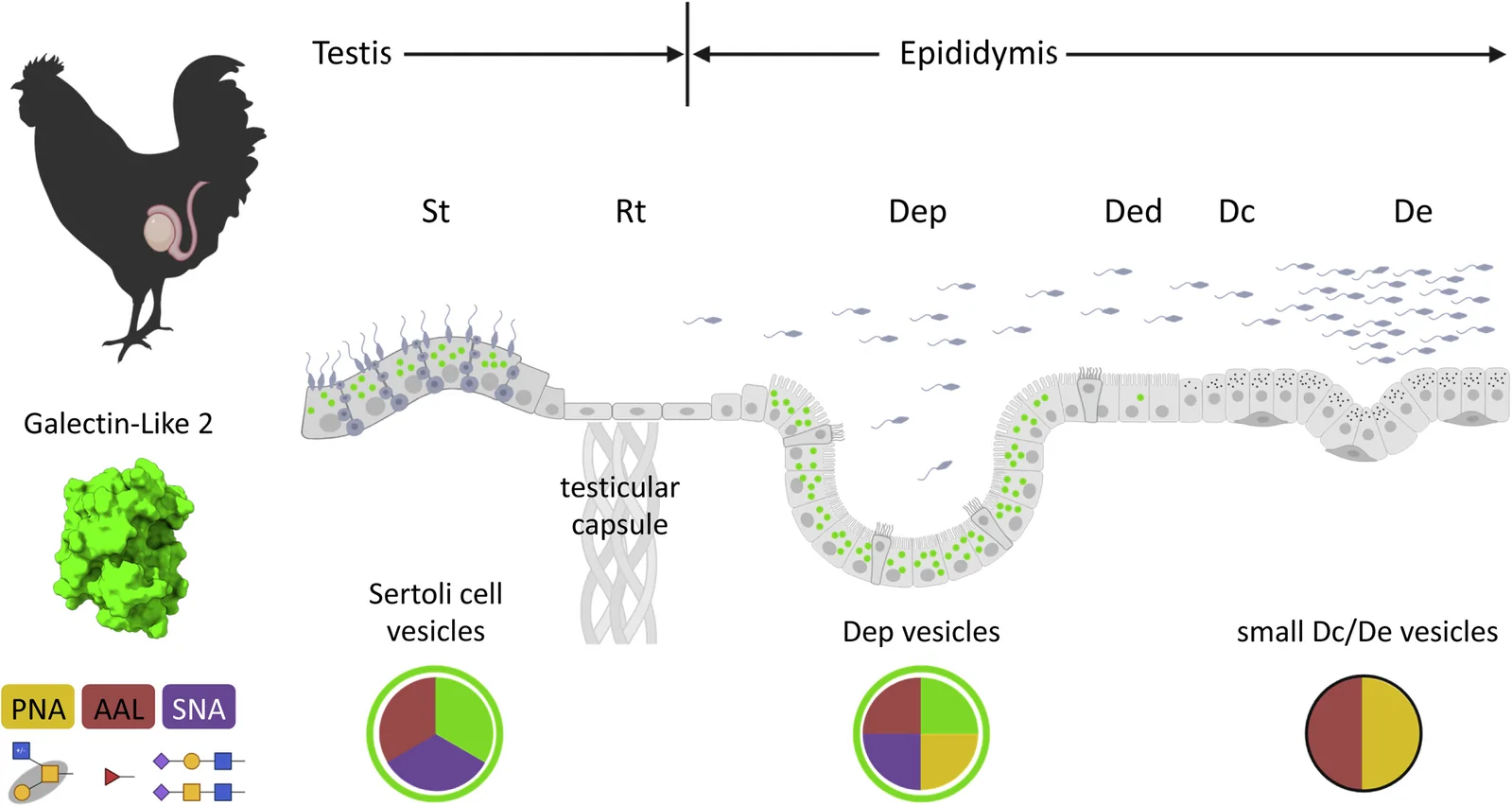
Graphical summary of the binding properties of carbohydrate-rich vesicles to galectin-like 2, as well as to PNA, AAL and SNA lectins, in the Sertoli cells in chicken testes and in the different epithelial cells that line the ducts of the epididymis. Dc, ductuli conjugetes; De, ductus epididymidis; Ded, ductuli efferentes distales; Dep, ductuli efferentes proximales; Rt, rete testis; St, seminiferous tubule. The diagram was created with BioRender.com. Not drawn to scale.

The efferent ducts of mammals are highly convoluted tubes which can account for up to 50% of the caput epididymis in certain species. In birds, the Ductuli efferentes, particularly in the proximal region, form large, convoluted sack-like structures with an increased surface area due to intense folding (Budras and Meier, 1981; Budras and Sauer, 1975). These ducts dominate the epididymis. The D. epididymidis itself, connected to the ductuli efferentes by the ductuli conjugentes, occupies a significantly smaller area. This contrasts starkly with mammals, where the epididymis, along with its subdivision into the caput, corpus and cauda regions, is significantly larger and more complex in structure. This is due to its essential role in sperm maturation. Since birds' sperm leave the testes at a more highly matured stage, it can be assumed that the function of the epididymis is less complex. Consequently, the passage of sperm through the efferent ducts and the subsequent change in the surrounding fluid is of paramount importance in birds. LGALSL2, which is absent in mammals, may also play a role here.

Given that the LGALSL2 protein binds to structures rich in glycans in galectin histochemistry, it was reasonable to assume that, despite its differences in the β-galactoside binding domain, the protein has glycan-binding properties. Investigating its binding to a large screening microarray containing 672 defined glycans should provide insight into which glycan-binding partners LGALSL2 might have. However, the analyses revealed only a few weak binding signals, mostly to fucosylated structures (Fig. 6A, Table S1). The majority of the signals were consistent with non-specific binding. Therefore, if LGALSL2 does possess glycan-binding activity, its ligands could be 'exotic' glycans, e.g. those expressed on mucin backbones, that are not included in this glycan probe set. A glycan microarray of the galectin-like protein chicken LGALSL, which is most similar to LGALSL2, showed that it only bound a few rather rare glycans, such as the 3′-sulphated Tn antigen, β-linked ribose, and the α-anomer of the TF antigen (García Caballero, et al., 2016). In the same study, binding and inhibition assays using glycoengineered CHO cells also showed no significant galectin-like interactions between chicken LGALSL with common mammalian-type glycans.

In addition to binding to potential unknown glycan ligands, the binding signals observed in LGALSL2 galectin histology could be due to mechanisms such as protein-protein interactions. This type of interaction is a well-known characteristic of galectin family members (Jacob and Gorek, 2024).

## Conclusion

LGALSL2 proteins are highly conserved members of the galectin family within the gnathostome lineage. While they exhibit strong homology to prototypical galectins, changes in the S-face groove result in a loss of β-galactoside binding function. Chicken *LGALSL2* is expressed in the male reproductive tract, with significantly higher expression levels in the testis than in the epididymis. Recombinant chicken LGALSL2 binds to glycan-rich vesicles in Sertoli cells in the testes and in epithelial cells in the D. efferentes proximales (Fig. 7). This suggests that the protein may play a role in the seminiferous fluid of the chicken, which is secreted by the Sertoli cells and resorbed by the efferent duct epithelium, and is important for sperm maturation. However, microarray screening tests carried out to identify possible glycan-binding partners of chicken LGALSL2 did not detect any relevant ligands under the conditions and the microarray sets used. Therefore, we can assume that LGALSL2 has rather 'exotic' glycan ligands or interacts via protein-protein interactions in testicular/epididymal tissue.

## Author contribution

Juliane Günther developed the study design. She carried out the phylogenetic and protein structure analyses and modelling, as well as the protein expression, purification and histochemical investigations. She also analyzed the data, wrote the initial manuscript, and reviewed and edited the final version. Antonio Di Maio conducted and analyzed the glycan microarray experiments, and reviewed and edited the manuscript. Alexander Rebl performed and analyzed the qPCR analyses, and also reviewed and edited the manuscript. Ralf Middendorff evaluated and interpreted the histochemistry data. In addition, he reviewed and edited the manuscript. Yan Liu conceptualized the microarray analyses, evaluated the data and reviewed and edited the manuscript. Sebastian P. Galuska was involved in the conceptualization of the study, the evaluation of the data, and the review and editing of the manuscript. All authors have approved the final manuscript for possible publication following peer review, and they personally vouch for the accuracy and integrity of the study results in all respects.

## Disclosures

The authors declare no conflicts of interest.

## Appendix Supplementary materials

Fig. S1: Histology of the adult chicken testis and the epididymis. (A) Masson trichrome staining illustrates the distribution of collagen and the structural organisation of the tissue. The collagen fibers in the testis capsule, interstitial tissue and lamina propria are shown in green, while the seminiferous tubule cells and the epithelial cells of the epididymal ducts are colored orange. The seminiferous tubules (St) of the testes, the rete testis (RT), the ductuli efferentes proximales (Dep) and distales (Ded), as well as the ductus epididymidis (De) and the ductuli conjugentes (Dc), are indicated. (B) PAS staining reveals the distribution of carbohydrate-rich structures in the testes and epididymis. Carbohydrates appear red. Nuclei are stained dark blue. The collagen fibres of the capsule, lamina propria and interstitial tissue, which appear green in trichrome staining, are also PAS-positive and appear red. Bv, blood vessel; Vc, vena cava.

Fig. S2: Lectin histochemistry of the chicken testis and epididymis. Tissue slides were incubated with biotinylated lectins. Binding was then detected using AF555-tagged streptavidin (green). The nuclei were counterstained with SYBR Green (blue). (A) PNA staining reveals intense binding signals in the D. epididymidis and the lumen of the D. efferentes distales (Ded). The epithelium of the D. efferentes proximales (Dep) shows weaker staining. Small blood vessels running through the epididymis are also stained. (B) In the control slides, co-incubation with 200 mM galactose inhibits PNA binding. (C) AAL stains the cells of the seminiferous tubules (St) of the testis. In the epididymis, the Dep epithelium and lumen show particularly strong staining. Staining is somewhat weaker in the distal regions (Ded, Dc and De). Connective tissue fibres are also stained. (D) Co-incubation with 400 mM fucose inhibits AAL binding to the slides. (E) SNA binds to the St cells. The Dep epithelium and lumen are also strongly stained. SNA staining is decreased in the epithelia of the distal efferent ducts. No SNA binding is detectable in the De epithelium. Cells of the interstitial tissue are stained too. (F) Neuraminidase pretreatment of the slides prevents SNA binding. (G) MAL-II shows weak binding in the St of the testis. Villi-like structures of the Dep epithelium and the lumen of these ducts are clearly stained. The epithelia of the De and Dc also show MAL-II staining. Marginal staining is present in the interstitium and the testicular capsule. (H) Pretreatment with neuraminidase enhances MAL-II binding, particularly to cells in the interstitium of the testis and epididymis, as well as to the testicular capsule.

Table S1: List of glycan probes in the broad spectrum glycan screening arrays and their binding scores (fluorescence intensity at 5 fmol/probe spot) elicited with LGALSL2 and AAL.

Table S2: Glycan microarray document based on MIRAGE Guidelines.

## Footnotes

Supplementary material associated with this article can be found, in the online version, at doi:10.1016/j.psj.2026.107952.
