# Supplementary material for: Galectin-like protein 2: A new non-mammalian member of the galectin family
Source: Poult Sci. 2026 Oct 11;105(12):107952. doi: 10.1016/j.psj.2026.107952 (PMC13696195; doi:10.1016/j.psj.2026.107952)
Supplement: Supplementary file 1 [file mmc4.docx]

MIRAGE DOCUMENT

**Table S2. Glycan microarray document based on MIRAGE Guidelines (doi:10.3762/mirage.3)**

| **Classification** | **Guidelines** |
| --- | --- |
| 1. **Sample: Glycan Binding Sample** | |
| Description of Sample | Sample names: Chicken LGALSL2 (biotin)  Origin: recombinant  Method of preparation:  Please see the Materials and Methods section in the main text.  Data of biotinylated *Aleuria Aurantia Lectin* (AAL) (VECTOR B1395) was included from comparison. |
| Sample modifications | Not relevant. |
| Assay protocol | please see Materials and Methods section in the main text. |
| **2.** **Glycan Library** | |
| Glycan description | The broad-spectrum screening glycan microarray contains 672 sequence-defined oligosaccharide probes. The probe names and corresponding structures are in Supplementary Table S2. These are a sub-set of a recently generated large screening microarray containing around 900 glycan probes (in-house designation ‘Array Sets 42-56’, which will be published elsewhere). The lipid-linked glycan probes are from the collection assembled in the course of research in the Glycosciences Laboratory (<https://glycosciences.med.ic.ac.uk/glycanLibraryList.html>). |
| Glycan modifications | For NGLs, unless otherwise specified these were prepared from reducing oligosaccharides by reductive amination with the amino lipid, 1,2-dihexadecyl-sn-glycero-3-phosphoethanolamine [(DHPE) ([Chai et al., Methods Enzymol. 2003](https://www.ncbi.nlm.nih.gov/pubmed/12968363))]; AO, NGLs prepared from reducing oligosaccharides by oxime ligation with an aminooxy functionalized DHPE [(AOPE) ([Liu et al., Chem. Biol. 2007](https://www.ncbi.nlm.nih.gov/pubmed/17656321))].  For full description on the definition of lipid moieties of the glycan probes please see <https://glycosciences.med.ic.ac.uk/docs/lipids.pdf> |
| 1. **3.** **Printing Surface; e.g., Microarray Slide** | |
| Description of surface | Nitrocellulose-coated glass microarray slides. |
| Manufacturer | 16-pad UniSart® 3D Microarray Slide from Sartorius (Goettingen, Germany) |
| Custom preparation of surface | Not relevant. |
| Non-covalent Immobilization | The lipid-linked probes were formulated as liposomes by adding carrier lipids, phosphatidylcholine (or 1,2-dihexanoyl-sn-glycero-3-phosphocholine, designated as DHPC) and cholesterol for robotically arraying and non-covalent immobilization on nitrocellulose-coated glass slides ([Liu et al., Methods Mol. Biol. 2012](https://www.ncbi.nlm.nih.gov/pubmed/22057521)). |
| **4. Arrayer (Printer)** | |
| Description of Arrayer | Nano-Plotter 2.1 (GeSim, Radeberg, Germany) |
| Dispensing mechanism | Non-contact liquid delivery with four dispensing tips. |
| Glycan deposition | Approximately 0.33 nl was printed per spot.  Lipid-linked glycan probes were printed at 2 and 5 fmol per spot in duplicate. |
| Printing conditions | The printing solutions were aqueous-based. Printing was performed at ambient temperature and relative humidity of 50-54%.  The printing solutions contained 100 pmol/μl each of cholesterol and DHPC as lipid carriers in addition to the lipid-linked glycan probes in water (HPLC grade). The concentrations of the lipid-linked glycan probes were 5 and 15 pmol/μl for the 2 and 5 fmol per spot levels, respectively. All printing solutions contained Cy3 NHS ester (GE Healthcare) at 20 ng/ml (26 fmol/μl) as a marker to monitor the printing process. |
| 2. **5.** **Glycan Microarray with “Map”** | |
| Array layout | Each array slide contained 16 subarrays (pads). Each pad was set up for printing 64 probes maximum, each at 2 levels in duplicate (four spots for one probe in a row); up to 256 spots (16x16) in total in each pad.  The 672 lipid-linked probes in the screening arrays were printed on multiple subarrays for parallel binding analyses. |
| Glycan identification and quality control | The quality control of the glycan microarrays was routinely carried out with (i) a panel of biotinylated plant lectins (Vector Laboratories), e.g. *Ricinus Communis* Agglutinin I (RCA120), *Aleuria aurantia* lectin (AAL), *Concanavalin A* (ConA) and wheat germ agglutinin (WGA), (ii) anti-carbohydrate antibodies, and (iii) commercial bacterial adhesins and toxins. Predicted binding results were recorded. These data will be published and shared via the GlyGen Glycan Array Repository currently under development as part of the NIH-funded GlyGen initiative (https://www.glygen.org/), which has entered its final testing phase. In the meantime, selected datasets can be seen in the shared Google folder via the link https://drive.google.com/drive/folders/1hMr-bWX4k3XxBd8FIB8cHbPkHBsxNzkj?usp=sharing.  AAL data are included in Figure 6 for comparison purposes. |
| 3. **6. Detector and Data Processing** | |
| Scanning hardware | GenePix 4300A (Molecular Devices, Berkshire, UK) |
| Scanner settings | Scanning resolution: 10 μm / pixel  Laser channel: Red (scan wavelength 635 nm)  PMT voltage: 350  Scan power: Adjusted for each sample to achieve maximum signal without saturation of any individual spot. The laser powers used for each sample are indicated in Supplementary Table S1. |
| Image analysis software | GenePix® Pro 7 (Molecular Devices, Berkshire, UK) was used for quantitation of the signal intensities from the array images. |
| Data processing | The gpr files were entered into an in-house microarray database using software (designed by Dr Mark Stoll, <http://www.beilstein-institut.de/en/publications/proceedings/glyco-2009>) for data processing. No particular normalization method, or statistical analysis was used. |
| **7.** **Glycan Microarray Data Presentation** | |
| Data presentation | The microarray binding results are presented as histogram charts in Figure 6. The results table with fluorescence intensity binding scores are in Supplementary Table S1 |
| 4. **8.** **Interpretation and** **Conclusion from Microarray Data** | |
| Data interpretation | No software or algorithms were used to interpret processed data. |
| Conclusions | Microarray screening analysis performed to search for potential glycan ligands of chicken LGALSL2 did not reveal significant binding under the conditions and with the array sets employed, in sharp contrast to the plant lectin AAL, which exhibited robust binding to fucosylated glycans. |
